# Clinical findings, treatment and outcome in cats diagnosed with precursor‐targeted immune‐mediated anaemia in a referral hospital in the UK: 30 cases (2010–2021)

**DOI:** 10.1002/vro2.70

**Published:** 2023-08-27

**Authors:** Alba Maldonado‐Moreno, Mayank Seth, Paola Monti, Rachel Miller

**Affiliations:** ^1^ Internal Medicine Service Dick White Referrals Ltd., Station Farm Cambridgeshire UK; ^2^ KGS Veterinary Services Limited Essex UK; ^3^ VCO Laboratory GmbH Hünenberg Switzerland; ^4^ CVS Referrals Vet Oracle Telemedicine Cambridge Cambridgeshire UK

## Abstract

**Aims:**

This retrospective study aimed to report clinical findings, treatment response and survival in cats diagnosed with precursor‐targeted immune‐mediated anaemia (PIMA) in a referral hospital in the UK.

**Methods:**

Feline cases diagnosed with presumed PIMA between January 2010 and February 2021 were retrospectively recruited. Signalment, clinical signs, diagnostic tests, treatment and outcome were recorded. Descriptive analytics were performed. Outcomes were documented according to survival to discharge, 30‐day survival, total survival time, response to immunosuppressive treatment and frequency of relapses.

**Results:**

Thirty cats met the inclusion criteria. A higher prevalence of females (19/30) was identified (*p =* 0.001). Most cats (25/30) presented with haematocrit below 0.15 L/L. Concurrent cytopenias occurred in 18 of 30 cats. Bone marrow diagnosis was erythroid hyperplasia in 24 of 30 cases. Survival to discharge was documented in 26 of 30 cats, of which 23 survived more than 30 days since diagnosis. Initial treatment included blood products (26/30) and prednisolone (26/30) or prednisolone with ciclosporin (3/30); 18 of 30 cats responded to treatment, with a normal haematocrit at a mean of 28 days. The initial haematocrit and the presence of concurrent cytopenia were not statistically different between responders and non‐responders. The median survival time was 140 days (range 1–3930 days).

**Conclusions and relevance:**

The treatment response rate of feline PIMA was high (60%), with a mortality rate of 23% over the 30 days following diagnosis. Relapses occur frequently (77%) but the response rate after treatment modification was high (76%) and therefore ongoing treatment may be justified at that point. Long survival times (up to 3930 days) can be achieved.

## INTRODUCTION

Feline precursor‐targeted immune‐mediated anaemia (PIMA) is an uncommon disease characterised by persistent non‐regenerative anaemia secondary to ineffective erythropoiesis.^1^ The terminology for this disease is controversial. Earlier terms include non‐regenerative immune‐mediated anaemia (NRIMA), non‐regenerative immune‐mediated haemolytic anaemia (NRIMHA) and pure red cell aplasia (PRCA), depending on the level of red cell destruction within the bone marrow (BM). It is unknown if PRCA, NRIMA and NRIMHA are separate conditions or if they represent different spectra of the same disease. In reports for dogs, the terms have been studied together under the term PIMA.^1^, ^2^

Published studies describing the clinical features and outcomes of cats with PIMA are limited. To date, the largest study included 15 cats,^3^ reporting a mortality rate of 27%. The same study reported that in cats responding to treatment, up to 6 weeks were required to achieve a normal haematocrit. Overall, little is reported regarding prognostic indicators in dogs and cats with PIMA.^1^, ^3^, ^4^

The aim of this study was to report clinical findings, survival, treatment and outcome in cats diagnosed with PIMA in the UK.

## MATERIALS AND METHODS

Laboratory records at a single multidisciplinary referral hospital were reviewed. Feline BM cytology and histopathology results obtained between January 2010 and February 2021 were identified. Cases with a diagnosis of erythroid hyperplasia, erythroid hypoplasia or pure red cell aplasia were eligible for inclusion. At the time of diagnosis, BM samples were microscopically evaluated (including a differential cell count). The BM samples were classified as erythroid hyperplasia when cellularity was normal or increased and the myeloid:erythroid (M:E) ratio was below 1.2.^5^, ^6^ Erythroid hypoplasia was defined as a normal or decreased BM cellularity with an M:E ratio above 2.16.^5^ Pure red cell aplasia was defined as a severe increase in the M:E ratio above 75:1 along with a non‐regenerative anaemia.^6^

Medical records were reviewed for those cases that had BM cytology and/or histology available. From these, cats with a diagnosis of PIMA were selected as potentially eligible for the study. Inclusion criteria for the study were as follows: presence of a persistent moderate to severe non‐regenerative anaemia (haematocrit below 0.25 L/L, reticulocyte count <60 cells × 10^9^/L) for more than 5 days, exclusion of other causes of non‐regenerative anaemia, compatible BM findings and a decision to start immunosuppressive therapy.

Cats were included in the study if complete medical records were available and if the following diagnostic investigations were performed: complete haematology (Siemens ADVIA 120 until 2018 and ADVIA 2120i Haematology System from 2019; Siemens Healthcare Diagnostics), blood smear assessment and manual platelet count, serum biochemistry (Olympus AU480 Chemical analyser; Beckman Coulter), thoracic radiography, abdominal ultrasound, feline immunodeficiency virus (FIV) and feline leukaemia virus (FeLV) serology (IDEXX SNAP FIV/FeLV Combo test) and BM sampling.

Cats with incomplete diagnostic investigations and/or medical records were excluded. Cats were also excluded when diagnosed with conditions that could lead to secondary non‐regenerative anaemia (such as FIV, FeLV, *Mycoplasma haemofelis*, *Candidatus Mycoplasma haemominutum* or *Candidatus Mycoplasma turicensis*, neoplasia, chronic kidney disease) or if they were receiving medications reported to cause adverse effects on the BM.^7^

The haematocrit at the time of presentation was recorded and cases were grouped as having severe anaemia (haematocrit <0.15 L/L) or moderate anaemia (haematocrit of 0.16–0.25 L/L; reference interval: 0.29−0.46 L/L).^3^, ^8^ When performed, the results of saline agglutination test and Coombs’ test were recorded. When performed, the results of real‐time PCR for FeLV provirus^9^ in BM were recorded. If not performed during the initial investigation, BM samples were retrospectively submitted for FeLV provirus PCR if available and viable.

Medical records were searched to collect data regarding immunosuppressive treatment choices, blood transfusions received, length of hospitalisation, survival to discharge, 30‐day survival and survival time. Survival times were calculated from the date of hospital admission. Follow‐up haematocrits and reticulocyte counts, treatment modifications (including discontinuation of immunosuppressive therapies) and relapses were also recorded. Cases were followed until death or until the end of the study period (February 2021). For those not re‐examined at the referral clinic, records and laboratory results were obtained from the referring veterinary surgeon.

Cases were classified as responders if they achieved a normal haematocrit. Response to treatment was also evaluated using: time to achieve regenerative response (reticulocyte count >60 cells × 10^9^/L), time to normalise haematocrit and time taken to discontinue immunosuppressive therapy. Cases were considered to relapse if they were reported to have a reduction in haematocrit requiring a dose increase or a reintroduction of immunosuppressive treatment. The time to relapse since initiation of treatment (or time between relapses), treatment received at relapse and any changes in the treatment due to the relapse were recorded.

In all cases, a signed consent form for the use of case data was provided by the owner at the time of initial hospitalisation. The study was ethically approved by Nottingham University (project number 2293 180516).

### Statistical analysis

Descriptive statistics were performed on all recorded data. Data were analysed using commercial statistical software (IBM SPSS Statistics for Macintosh, version 28.0). Variables were assessed for normality by histograms and the use of Shapiro–Wilks tests. The continuous data were expressed using medians and ranges and were compared with the Mann–Whitney *U*‐test. Significance was defined as *p*‐value 0.05 or less. Survival was assessed by Kaplan–Meier product limit estimates.

## RESULTS

Over the study period 77 cats underwent BM sampling. Of these, 37 had persistent non‐regenerative anaemia and BM analysis identified erythroid hyperplasia, erythroid hypoplasia or red cell aplasia without evidence of an underlying cause.

Seven cats (7/37) were excluded from the study, of which four were females and three were males. Of these, three received BM suppressive drugs over the previous 3 months (carbimazole [1/37], methimazole [1/37] and chlorambucil [1/37]). Three cats were excluded due to a high suspicion of neoplasia (intestinal mass [2/37] and pulmonary nodules [1/37]). One cat was excluded because it tested positive for proviral FeLV by PCR (1/37). Thirty cats with a presumptive diagnosis of PIMA met the final inclusion criteria.

### Signalment and clinical presentation

There were 19 females (63%; all neutered) and 11 males (36%; 10 neutered and one entire). During the study period, the feline clinic population comprised a total of 8942 cats. Therefore, the prevalence of PIMA during that time was 0.003. The feline population comprised 3892 females (43%) and 5050 males (56%); the female:male ratio was 0.77:1.0. Precursor‐targeted immune‐mediated anaemia affected a greater proportion of female cats in the study group, which was statistically significant (*p* = 0.01). Of the study population, 27 of 30 (90%) cases were domestic shorthaired cats. Three cats were pedigree breeds: one Siamese, one Maine Coon and one Burmilla. The median age at diagnosis was 24.3 months (range 7−158 months). Presenting clinical signs included lethargy (27/30; 90%), anorexia (25/30; 83%), weight loss (12/30; 40%), pica (10/30; 33%), collapse (5/30; 16%) and polyuria and polydipsia (2/30).

### Clinicopathological findings

Haematology and biochemistry results are summarised in Table 1. Urinalysis was performed in seven of 30 (23%) cats and no abnormalities were reported. Severe anaemia (haematocrit <0.15 L/L) was detected in 25 of 30 (83%) cats. Concurrent cytopenias were reported in 18 of 30 (60%) cats: 16 (53%) cats were bicytopenic and two (6%) were pancytopenic. Of the 16 bicytopenic cats, nine (56%) cats were thrombocytopenic (mean platelet count of 88 cells × 10^9^/L; range 5–180 cells × 10^9^/L) and seven (43%) cats were neutropenic (mean neutrophil count of 1.4 cells × 10^9^/L; range 0.9–2.3 cells × 10^9^/L). Both cats with pancytopenia showed combined neutropenia (neutrophil count of 1.2 cells × 10^9^/L and 1.32 cells × 10^9^/L) and thrombocytopenia (platelet count of 100 cells × 10^9^/L and 110 cells × 10^9^/L).

**TABLE 1:** Haematology and biochemistry results at admission of 30 cats with precursor‐targeted immune‐mediated anaemia (using reference intervals [RI] from DWR Laboratory Diagnostics).

	Variable (unit)	Median	Range	Cats with values <RI	Cats with values within RI	Cats with values >RI	Reference interval
Haematology	Haematocrit (L/L)	0.10	0.03−0.21	30	0	0	0.29−0.46
	Mean corpuscular volume (fL)	53.1	29.2−85.2	3	12	15	37−52
	Reticulocyte count (×10^9^/L)	22.9	0.9−51	N/A	30	0	0−60
	White blood count (×10^9^/L)	10.32	2.9−40.1	5	19	6	5.5−19.5
	Neutrophils (×10^9^/L)	4.13	0.9−36.7	7	20	3	2.5−12.5
	Lymphocytes (×10^9^/L)	3.36	0.7−17.2	4	30	6	1.5−7.0
	Monocytes (×10^9^/L)	0.42	0−3.6	N/A	24	6	0.0−1.0
	Eosinophils (×10^9^/L)	0.09	0−1.8	N/A	29	1	0.0−1.5
	Platelets (×10^9^/L)	272	5−593	11	19	0	250−800
Biochemistry	Albumin (g/L)	30	24−37	0	30	0	24−39
	Globulins (g/L)	41	30−74	0	20	10	20−45
	Urea (mmol/L)	9.5	3−17.4	4	17	9	6−10
	Creatinine (mmol/L)	97.5	47−208	0	26	4	40−140
	Alanine aminotransferase (IU/L)	59.5	10−331	1	20	9	30−85
	Alkaline phosphatase (IU/L)	15	4−80	0	29	1	4−62
	Total bilirubin (mmol/L)	6.5	0−132	N/A	21	9	0−10

Abbreviation: N/A, not applicable.

Globulin levels were elevated in 10 cases (10/30): nine cats had a mild globulin elevation (between 46 and 61 g/L; range 20–45 g/L) and one cat had a marked elevation of 76 g/L. The cat with marked hyperglobulinaemia was negative for *M. haemofelis* PCR and *Feline Coronavirus* serology; it was documented to have normal globulin levels after 1 month of treatment.

Four cats (4/30, 16%) had elevations in both urea and creatinine on admission. The creatinine normalised in follow‐up samples of all those cats and the urine specific gravity was greater than 1.040 in all cases; therefore, the azotaemia was considered pre‐renal. Five additional cats (5/30, 16%) had elevated urea with a normal creatinine concentration. No clinicopathological variables were significantly associated with 30‐day survival.

Cobalamin was within normal limits in all three of 30 (10%) cats in which it was measured. Coombs’ test was performed in six of 30 (20%) cases, of which four were positive and two were negative. Agglutination tests were performed in 10 of 30 (33%) cases: four (40%) were positive and six (60%) were negative.

Of the 30 cats in the study, FeLV provirus PCR was performed for 27 (90%) cats. The test was performed either at the time of diagnosis (in 15/27 [55%] cats) or retrospectively (in 12/27 [45%] cats). All of the cats tested (100%) were negative. In three of 30 (10%) cats, the slides were no longer available and, therefore, FeLV provirus PCR could not be performed. Haemotropic mycoplasma PCR in blood was performed in 13 of 30 (43%) cases and was negative in all the tested cats (100%). A total of 17 of 30 (57%) cats were not tested for haemotropic mycoplasma.

Thoracic radiography identified mild cardiomegaly in nine of 30 (30%) cases. Of those nine cases, echocardiography was performed in five of 30 (16%), which did not identify evidence of cardiomyopathy. Abnormalities detected on abdominal ultrasonography included mild lymphadenomegaly (6/30; 20%), mild hepatomegaly (3/30; 10%), splenomegaly (7/30; 23%) and mild abdominal effusion compatible with a protein poor transudate (3/30; 10%). Fine‐needle aspirates were obtained from all six cats with lymphadenomegaly (all consistent with reactive lymphoid hyperplasia), one cat with hepatomegaly (consistent with cholestasis) and three of the cats with splenomegaly (two were consistent with extramedullary haematopoiesis and one with reactive lymphoid hyperplasia).

### Bone marrow findings

Nine cats (30%) had only BM cytology submitted; four of 30 (13%) had only a BM core biopsy; and 17 of 30 (57%) had both cytology and core. Of 30 cats, bone marrow analysis was compatible with erythroid hyperplasia in 24 (80%) cats, erythroid hypoplasia in four (13%) cats and pure red cell aplasia in two (6%) cats. The details of the BM results are presented in Table 2.

**TABLE 2:** Results of the bone marrow examination from cats with precursor‐targeted immune‐mediated anaemia.

	Total study population (Range), *n* = 30/30	Erythroid hyperplasia (Range), *n* = 24/30	Erythroid hypoplasia (Range), *n* = 4/30	Red cell aplasia, *n* = 2/30	Reference interval
Lymphocytes (%)	7 (0−47.5)	6.75 (0−23.5)	7.5 (4.7−7.5)	44 and 47.5	5.6−16
Total myeloid cells (%)	15 (5−74)	14 (5−57)	72 (72−74)	42 and 47	
Total erythroid cells (%)	77 (1−94)	81 (41−94)	17 (17−21)	1 and 4	
Myeloid: erythroid ratio	0.2 (0.06−47)	0.2 (0.06−1.07)	3.88 (3.5−4.2)	10.5 and 47	1.2−2.2
Myelofibrosis	4/30 cats	4/30 cats	0/30 cats	0/30 cats	

*Note*: All numbers out‐with brackets represent mean values unless stated otherwise.

In cases undergoing core biopsy sampling, myelofibrosis was reported in four of 21 (19%) cases. All cats with myelofibrosis presented with erythroid hyperplasia and mild myelofibrosis, which was considered a secondary change. Rubriphagocytosis was observed in four of 30 (13%) cats.

### Treatment

No cats received blood products or immunosuppressive medication before admission. Blood products were administered in 26 of 30 (86%) cats at presentation. Of 26 cats, a single transfusion was given in 14 (54%) cats, two transfusions were used in 11 (42%) cats and one (4%) cat required three transfusions. The details on the blood transfusions administered are reported in Table 3.

**TABLE 3:** Type and number of blood transfusions administered to cats with precursor‐targeted immune‐mediated anaemia.

	One transfusion event, *n* = 14/30 cats	Two transfusion events, *n* = 11/30 cats	Three transfusion events, *n* = 1/30 cat
Feline whole blood only	9	2	0
Feline packed red blood cells (pRBC) only	2	3	1
Oxyglobin only	3	4	0
Oxyglobin + feline whole blood	0	1	0
Whole blood + pRBC	0	1	0

Along with transfusions (if clinically required), cats were started with prednisolone as a single immunosuppressive (26/30; 86%) or prednisolone combined with ciclosporin (3/30; 10%). The median prednisolone dosage was 2.5 mg/kg once daily (range 1.6−3.75 mg/kg once daily). The ciclosporin dosage ranged between 4.7 and 9 mg/kg once daily. The cat with a haematocrit of 0.03 L/L did not receive immunosuppressive treatment (although planned) because it had a cardiopulmonary arrest shortly after a second transfusion.

Antiplatelet medications were prescribed in two of 30 (6%) cats (clopidogrel [one] and aspirin [one]). Doxycycline was administered with prednisolone in seven of 30 (23%) cats pending *Mycoplasma* PCR results. No cats received gastroprotectants.

### Outcome

**FIGURE 1:**
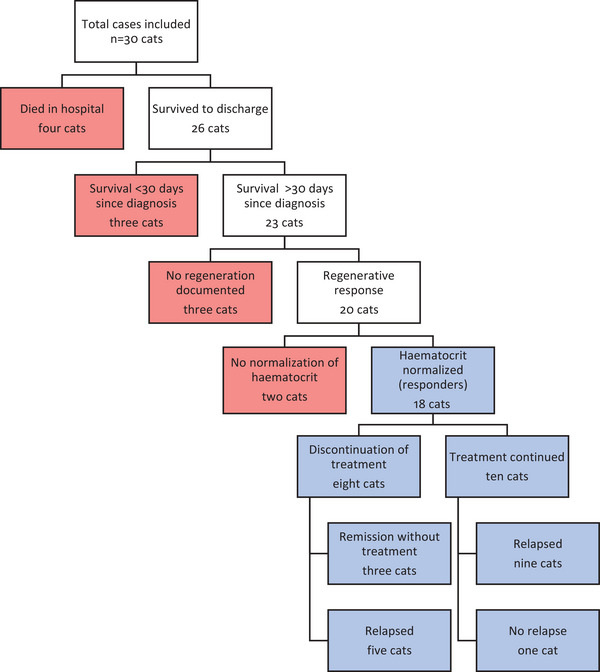
Flow chart of case progression of cats included in the study. Red boxes indicate cats classified as non‐responders. Blue boxes indicate cats classified as responders.

A summary of the case outcomes and progression is provided in Figure 1.

### Short‐term outcome

The median hospitalisation time was 7 days (range 1−18 days). Four cats did not survive to discharge: two had cardiopulmonary arrest at days 1 and 4 post‐diagnosis, and two were euthanased at days 4 and 6 after diagnosis. Of the 26 cats discharged from hospital, three were euthanased within 30 days from diagnosis due to relapsing anaemia (at days 12, 17 and 18 post‐diagnosis). Of the 23 cats surviving more than 30 days, four cats did not receive any blood transfusion, 11 cats received one blood transfusion, seven cats received two blood transfusions and one cat received three blood transfusions.

Of the 23 cats surviving longer than 30 days, three cats never showed a regenerative response. All three cats received a single blood product transfusion and prednisolone. Twenty cats developed a regenerative response and the median time to observe regeneration was 13 days after starting treatment (range 5−63 days). Eighteen cats had a normal haematocrit at a median of 28 days (range 16−226 days) and were classified as responders. Two of the cats initially showed signs of regeneration but did not reach a normal haematocrit; in one cat the haematocrit remained below 0.16 L/L (without signs of haemolysis or bleeding) and was euthanased 54 days post‐diagnosis. A second cat had a haematocrit of 0.25 L/L but was euthanased after relapsing 78 days post‐diagnosis.

### Responding cats

Within the 18 responder cats that had a normal haematocrit, the reported treatments at the time of normalisation of haematocrit were prednisolone (17/18; 94%; dosage 1.6−3.75 mg/kg once daily) and prednisolone with ciclosporin (1/18; 5%; dosage 5 mg/kg once daily). The initial haematocrit and evidence of concurrent cytopenias were not statistically significant between responders and non‐responders.

Immunosuppressive treatment was stopped in eight of 18 (44%) of the responders at a median of 200 days after starting treatment (range 68−397 days). Three cats remained in remission without treatment and were alive at the end of data collection (survival 251−2361 days). The remaining five of 18 (27%) cats relapsed at a median of 224 days after treatment withdrawal (range 31−500 days).

### Relapses

Of the responders, disease relapse was reported in 14 of 18 cats (77%). At the time of relapse, nine of 14 (64%) cats were receiving treatment. The median time from starting treatment to relapse was 224 days (range 99−980 days).

One cat (1/14) was euthanased at relapse with no treatment modification. The treatment was modified in 13 of 14 (92%) relapsing cats and 10 of 13 cats (76%) responded. Reported treatment modifications included prednisolone alone (9/14; 64%; dosage 2 mg/kg once daily) or prednisolone combined with ciclosporin (1/14; 7%; dosage 13 mg/kg once daily), chlorambucil (1/14; 7%; dosage 2 mg/m^2^ every 48 h) or mycophenolate (2/14; 14%; dosage 12 mg/kg twice daily).

### Survival time

**FIGURE 2:**
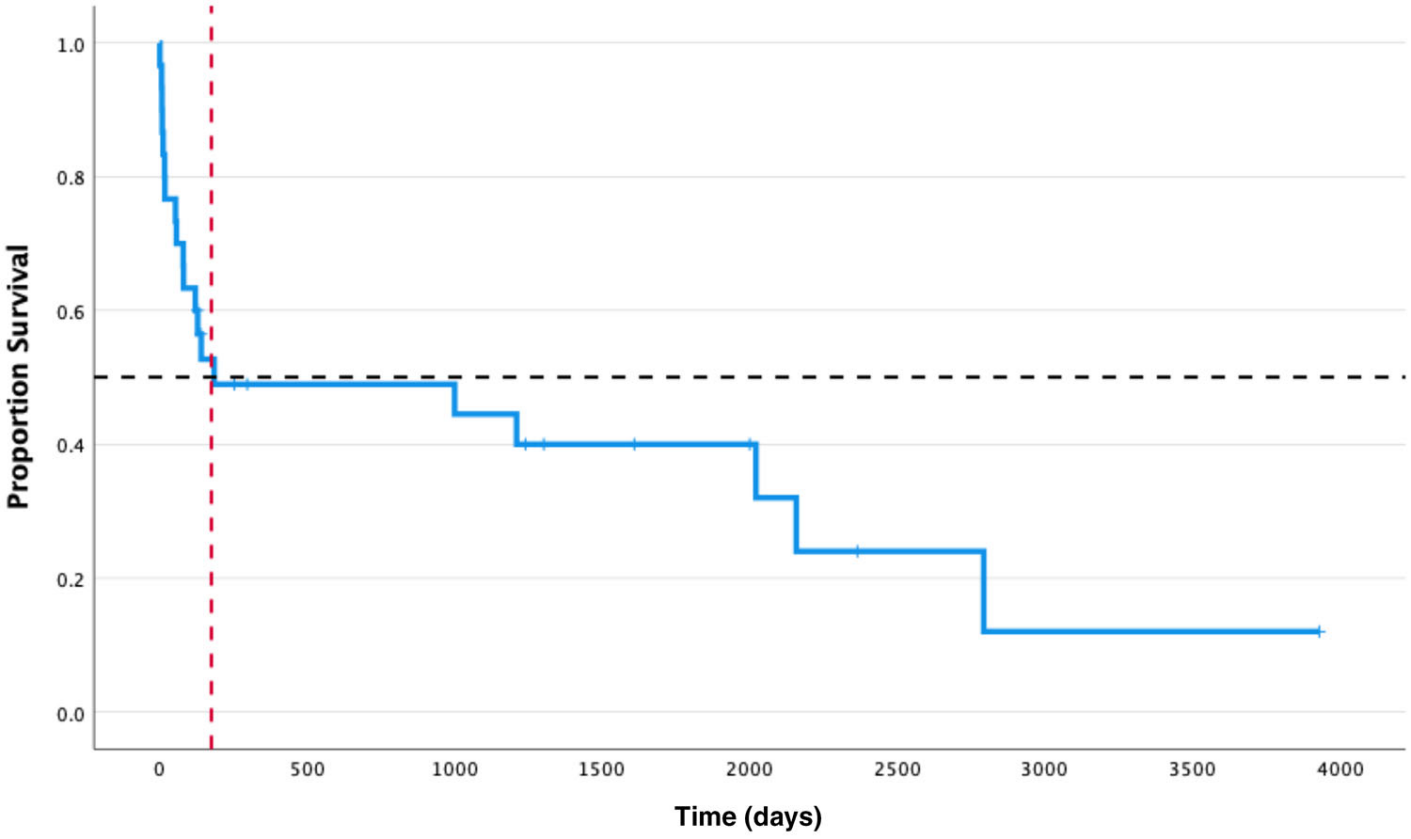
Kaplan–Meier survival curve showing the survival times of cats diagnosed with precursor‐targeted immune‐mediated anaemia (blue line). Tick marks indicate censored cases (cats alive at the end of the study period). The black dashed line indicates the median value in the proportion of survival axis and the red dashed line indicates the median survival time (days). Median survival time was 140 days.

The median survival time was 140 days since diagnosis (range 1−3930 days). Ten cats (10/30; 33%) were alive at the end of the study (Figure 2) with a median follow‐up time of 1271 days (range 128−3930 days). Within the study period, of 30 cats, 18 (60%) cats died from PIMA‐related causes and two (6%) cats died from causes seemingly unrelated to PIMA: one cat was involved in a road traffic accident 121 days post‐diagnosis and one cat developed hyperthyroidism and congestive heart failure 2158 days (5.9 years) post‐diagnosis.

## DISCUSSION

This retrospective study describes the clinical presentation and outcomes of 30 cats diagnosed with PIMA in a referral clinic over 11 years. Our data showed that the prevalence of this condition in our clinic was low (0.003) and that there was a significantly higher prevalence of PIMA in females (19/30 cases, 63%), which is in contrast with a previous study in 15 cats with PIMA in which five of 15 (33%) of included cases were female.^3^ Both studies had small populations, which may have affected this result. However, our clinic's sex distribution (43% females) differed from the reported feline distribution in the UK^10^; therefore, comparison of our sex distribution data with other studies may not be possible. In dogs with PIMA, females were over‐represented in the population of two previous studies.^4^, ^11^

In our study, cats were considered to have a non‐regenerative response if the total reticulocyte count was below 60 cells × 10^9^/L based on the internally validated upper reference range of our institution's laboratory for cats. This internal validation was made in accordance with the American Society for Veterinary Clinical Pathology Quality Assurance and Laboratory Standards Committee guidelines for establishing de novo veterinary reference intervals.^12^ Previous studies have reported the same value as reference interval for total reticulocyte count.^6^ However, direct comparison of our results with previous studies may not be possible because reference intervals and definitions for feline non‐regenerative response vary across previous reports.^3^, ^8^, ^13^

The majority of cats were severely anaemic (median haematocrit of 0.10 L/L) on presentation, similar to a previous report.^3^ The median initial haematocrit in our study was similar between responders and non‐responders, indicating that in our study this parameter did not provide prognostic information. Macrocytosis (15/30 cats) and leukocytosis (6/30 cats) were frequent. Concurrent cytopenias were frequent (60% cats). They were mostly moderate, with only one cat having severe thrombocytopenia below 50 cells × 10^9^/L and one cat showing marked neutropenia below 1 cell × 10^9^/L. Although the prevalence of concurrent cytopenias has been described as high (55/72; 76%) in cats with primary IMHA,^13^ their frequency was previously unreported for cats with PIMA. Two studies in dogs with PIMA documented a prevalence of 41%^1^ and 22%.^4^

Hyperbilirubinaemia has been identified as a negative prognostic factor in canine and feline IMHA, as well as in canine PIMA.^14^, ^15^ In our study, hyperbilirubinaemia was detected in nine of 30 (30%) cats, all of which survived longer than 30 days. Seven of the responders (7/18, 38%) were hyperbilirubinaemic at presentation. This suggests that hyperbilirubinaemia might not be associated with worse prognosis in cats with PIMA.

Erythroid hyperplasia accounted for the majority of BM diagnoses in our population (24/30 cats; 80%), which is a higher proportion than previously reported in cats^3^ and dogs.^4^ A high prevalence of rubrophagocytosis has been reported in canine PIMA,^1^, ^4^ although in our population, it was documented in four of 30 cats. This finding is in line with that previously documented in feline PIMA.^3^ Myelofibrosis was documented in four of 22 cats and was considered mild in all cases. Two of the cats with myelofibrosis had long survival times of 1186 and 2012 days, respectively, suggesting that in common with previous reports,^1^, ^3^, ^4^ mild myelofibrosis might not be associated with a worse prognosis.

The majority of our cases required blood transfusions and were treated with prednisolone as a first‐line treatment, as previously described.^3^, ^14^, ^15^ Second‐line immunosuppressives used in our population included ciclosporin, chlorambucil and mycophenolate mophetil. The use of ciclosporin and chlorambucil has been described in feline PIMA,^3^, ^16^ although mycophenolate has been reported in cats with IMHA^13^ and dogs with PIMA,^1^ to the best of the authors’ knowledge, this is the first report of the use of mycophenolate combined with glucocorticoids for feline PIMA. In our population, the two cats treated with mycophenolate failed to increase the haematocrit and were euthanased 25 and 44 days later (total survival 129 and 185 days, respectively). No gastrointestinal side effects associated with mycophenolate were reported in our population. The reasons for introduction of a second immunosuppressant and the choice of agent were not clear, given the retrospective nature of this study. The low numbers of cases treated with second agents in this and other studies make it challenging to draw conclusions on the potential benefit, as is the case with other immune‐mediated diseases in cats and dogs.^15^

Only two of 30 (6%) cats included in our study received antithrombotics. To date, no data have been published supporting the use of antithrombotics in feline PIMA and the prevalence of thromboembolic events in this condition remains unknown.^3^, ^16^, ^17^ Antithrombotics are recommended in the treatment of dogs with primary IMHA,^18^ with one report supporting their use in cases of canine PIMA.^1^

A response rate of 60% (18/30 cats) was observed in our population. This is lower than that previously reported.^3^ Despite the high response rates, the prevalence of relapses was also high in our population: 77% of cats relapsed once and 42% of cats relapsed twice. The relapse rate was higher than that previously reported in cats with PIMA^3^ (3/11 cats; 27%) and in cats with primary IMHA^8^ (5/16 cats; 31%). Although relapses were frequent, the majority of cats responded to treatment modification. A previous study in feline PIMA^3^ reported similar findings, with all three of 15 relapsing cats responding to treatment modifications. These data suggest that relapse is not necessarily associated with poorer survival and that ongoing treatment attempts are justified.

The current study had several limitations. The low number of recruited cases limited interrogation of clinicopathological prognostic factors (such as hyperbilirubinaemia) and treatment comparisons. We believe that obtaining information about prognostic factors would be clinically relevant to identify cases that are less likely to respond to treatment or that could require more intensive treatment. Our population was heterogeneous, including cats with different BM findings. The impact of these differences in the outcome is unknown, although performing BM is still useful to exclude other BM conditions leading to anaemia.

Some limitations were inherent to the retrospective design of the study. Decisions regarding additional diagnostic investigations (such as Coombs’ test and *Haemoplasma* spp. PCR) and treatment choice were not standardised. None of the cases had iron levels measured to investigate for anaemia of iron deficiency and vitamin B12 concertation was not consistently assessed. Azotaemia was evaluated based on increased creatinine only, this, it is not possible to completely exclude chronic kidney disease in the five included cats with elevated urea and normal creatinine.

In summary, the majority of cats with PIMA responded to immunosuppressive treatment, although relapses were frequent. Long survival times can be achieved. Further studies are warranted to identify prognostic factors and to compare treatment protocols.

## AUTHOR CONTRIBUTIONS

All authors contributed to the planning, execution and writing of this manuscript.

## CONFLICTS OF INTEREST STATEMENT

The authors declare they have no conflicts of interest.

## FUNDING INFORMATION

The authors received no specific funding for this work.

## ETHICS STATEMENT

The authors confirm that the ethical policies of the journal, as noted on the journal's author guidelines page, have been adhered to. The research was approved by the ethical review committee at Nottingham University (ethical approval number 2293 180516).

## Data Availability

The data that support the findings of this study are available from the corresponding author upon reasonable request.
